# Integration of intraoperative ultrasound and depth-electrode electrocorticography for resection guidance in epilepsy surgery: technical workflow and feasibility

**DOI:** 10.1007/s00701-026-06792-9

**Published:** 2026-02-16

**Authors:** Luca Zanuttini, Elena Pasini, Lorenzo Ferri, Lidia Di Vito, Anna Scarabello, Francesca Bisulli, Matteo Martinoni

**Affiliations:** 1 Department of Neurosurgery, IRCCS Istituto Delle Scienze Neurologiche Di Bologna (ISNB), Bellaria Hospital, Via Altura 3, 40139 Bologna, Italy; 2 Department of Biomedical and Neuromotor Sciences (DIBINEM), University of Bologna, Bologna, Italy; 3 Epilepsy Program, Full Member of European Reference Network EpiCARE, IRCCS Istituto Delle Scienze Neurologiche Di Bologna, Bologna, Italy

**Keywords:** Case series, Depth electrodes, Electrocorticography, Epilepsy surgery, Intraoperative ultrasound, Neurophysiological monitoring

## Abstract

**Background:**

Complete resection of the epileptogenic zone (EZ) is the strongest predictor of seizure freedom in drug-resistant epilepsy (DRE). However, even in MRI-positive cases with anatomo-electro-clinical concordance, the EZ may not be clearly delineated, complicating intraoperative decision-making. Intraoperative ultrasound (ioUS) provides real-time anatomical feedback, while depth-electrode intraoperative electrocorticography (iECoG) enables electrophysiological delineation of epileptogenic tissue beyond the cortical surface, sampling deep regions not accessible to subdural electrodes. Their integration may improve intraoperative precision in defining resection limits and optimizing resective surgery.

**Methods:**

This study describes the workflow and feasibility of combining ioUS and depth-electrode iECoG for intraoperative guidance in MRI-positive focal DRE with an ill-defined EZ. In all cases, concordant anatomo-electro-clinical data identified a single EZ for which SEEG was not required, yet the suspected EZ remained poorly delineated. ioUS was used for real-time lesion visualization, verification of electrode trajectories, and guidance of resection depth and extent. Pre- and post-resective depth-electrode iECoG and ioUS were used in combination to delineate the resection margins, by identifying interictal epileptiform discharges (IEDs) and confirming lesion boundaries and resection completeness.

**Results:**

Six patients underwent resective surgery using the combined ioUS–iECoG workflow. The technique was feasible and safe in all cases, with no intraoperative or postoperative complications (37 depth-electrode insertions). iECoG revealed IEDs in four patients (66%), prompting resection extension in two. MRI confirmed complete resection in all cases. At last follow-up (6–40 months), 5/6 patients were seizure-free (Engel I). Histopathology revealed FCD IIb in three cases, a gliotic lesion related to an encephalocele in one, a gliotic scar post–arachnoid cyst marsupialization in another, and a tuberous sclerosis–related lesion in a case of tuberous-sclerosis-complex.

**Conclusion:**

The integration of ioUS and depth-electrode iECoG offers real-time anatomical and electrophysiological data, refining EZ delineation and resection assessment in complex MRI-positive epilepsy cases where SEEG is not clinically indicated.

## Introduction

The prevalence of drug-resistant epilepsy (DRE) among persons with epilepsy (PWE) remains a significant clinical challenge, with approximately 30% of PWE affected [20]. For patients with focal DRE, surgical resection of the epileptogenic zone (EZ) offers the most effective treatment, with the potential to achieve seizure freedom or substantial seizure reduction [26, 30]. Surgical success critically depends on accurate delineation and complete resection of the EZ.

In MRI-positive cases, the epileptogenic lesion may not perfectly overlap with the EZ, making complete resection challenging [6]. Incomplete resection or inaccurate localization of the EZ are the main causes of postoperative seizure recurrence, motivating extensive efforts to refine pre- and intraoperative methods for EZ identification [2, 9, 19, 25]. These include high-resolution MRI, PET, and SPECT with advanced post-processing, as well as invasive monitoring using subdural grids or stereo-EEG (SEEG). When SEEG is not performed, alternative approaches often fail to provide the surgeon with reliable intraoperative guidance on the true boundaries of the EZ. This limitation is particularly evident in deep-seated lesions and in those with indistinct margins [23]. Consequently, increasing reliance has been placed on real-time intraoperative tools to refine surgical strategies and enhance EZ identification.

Intraoperative electrocorticography (iECoG), first introduced by Penfield and Jasper in the 1930s [4], has been widely used in epilepsy surgery until the development of tools for pre-resective invasive electrophysiological monitoring like subdural grids and SEEG. These techniques overcame the spatial and temporal limitations of iECoG in identifying interictal epileptiform discharges (IEDs) [18]. In recent years, new insights on peculiar electrophysiological activity of epileptogenic lesions like Focal Cortical Dysplasia (FCDs) [8, 16], together with technical development of new iECoG probes and research on new interictal biomarkers, like high frequency oscillations (HFOs) [28], shed a new light on iECoG-guided surgery. Recent reviews highlighted its impact on seizure outcome [15, 17].

To date, iECoG is most commonly performed using electrode strips or grids. Depth electrodes, commonly used in SEEG procedures, constitute a valid alternative, particularly in deeply located lesions like bottom-of-sulcus dysplasias [11]. However, their intraoperative application is often hindered by limited information about electrode placement and the anatomical structures explored.

The combined use of intraoperative ultrasound (ioUS) and depth-electrode iECoG has been recently proposed to address these limitations, yet evidence is limited to isolated reports or single technical notes [5, 21, 27]. The role of ioUS in epilepsy surgery has been reinforced by recent studies describing the sonographic features of FCDs, together with the well-known features of tumoral lesions [2, 3, 12, 22, 24, 27]. By integrating real-time anatomical visualization with electrophysiological mapping, depth-electrode iECoG-ioUS-guided surgery could enhance the surgeon's ability to localize and resect the EZ with precision. ioUS provides real-time delineation of lesion boundaries, while its integration with the depth electrode ensures accurate localization of recording contacts, serving as landmarks for resection extent based on electrophysiological findings.

We therefore present the first structured description of a real-world workflow integrating depth-electrode iECoG and ioUS for resective epilepsy surgery, illustrated through a consecutive case series of MRI-positive DRE patients. Our aim is to demonstrate the feasibility, reproducibility, and potential clinical value of this combined intraoperative guidance strategy.

## Materials & methods

### Patient selection

We report a retrospective consecutive series of selected patients with drug-resistant epilepsy who underwent tailored resection guided by intraoperative depth-electrode electrocorticography and ultrasound (ioUS) at our institution between 2022 and 2024. All included patients presented with MRI-visible lesions and concordant anatomo-electro-clinical findings consistent with a single epileptogenic focus, for which SEEG investigation was not deemed necessary. Although the EZ could be defined preoperatively, its anatomical boundaries remained uncertain, particularly in cases of suspected FCD or glioneuronal tumors (GNTs) with an associated dysplastic component, or were considered difficult to tailor intraoperatively due to deep or sulcal involvement and/or proximity to eloquent cortex. Accordingly, intraoperative guidance was complemented by depth-electrode iECoG and ioUS, with the specific aim of refining the anatomical definition of resection limits. In all cases, conventional subdural strip iECoG was also employed; however, lesion morphology and sulcal or subcortical extension limited its contribution to intraoperative decision-making, whereas depth-electrode recordings proved more informative for functional assessment and tailoring of resection.

### Presurgical evaluation

MRI was acquired according to the HARNESS protocol, including coronal 2D T2-weighted, 3D FLAIR, and 3D T1-weighted sequences [7]. All patients underwent prolonged video-EEG monitoring to establish anatomo-electro-clinical correlations. In each case, interictal [21]F-FDG PET was performed to identify areas of cortical hypometabolism concordant with MRI and clinical data.

Advanced neuroimaging post-processing (FreeSurfer, FSL, MRIcroGL, and 3D Slicer) was used to improve anatomical contextualization of MRI-visible abnormalities and to support pre-surgical planning [10]. In cases with suspected focal cortical dysplasia, the Morphometric Analysis Program (MAP) was also applied [29]. Each case was discussed within a multidisciplinary team including epileptologists, neuroradiologists, and neurosurgeons involved in both diagnostic and therapeutic phases.

Electrode trajectories and planned resection boundaries were defined within the 3D Slicer platform and transferred to the neuronavigation system (StealthStation S8, Medtronic) for intraoperative guidance. The number and orientation of planned trajectories were tailored on a case-by-case basis according to lesion extent, depth, and morphology to ensure adequate coverage of the suspected epileptogenic tissue. After resection, a depth electrode was positioned along the cavity margins under ioUS guidance, targeting regions comparable to the pre-resection sampling trajectories. No additional pre-resection electrodes or unplanned trajectories were created intraoperatively.

### Surgical workflow

An initial panoramic ioUS scan was obtained (Esaote MyLab, multifrequency micro-convex probe, 3–11 MHz) before dural opening to identify the lesion and assess its echogenic features. After dural opening, pre-resective electrophysiological sampling was performed by introducing a depth electrode along the preplanned trajectory to the lesion margin, under combined neuronavigation and real-time ioUS guidance.

Depth electrodes (Dixi Medical, Besançon, France) with either 5 or 8 contacts were used. The active contact column measured approximately 16 mm in the 5-contact model and 26.5 mm in the 8-contact model. Before craniotomy, additional scalp electrodes were placed at Cz (or Pz), contralateral homologous regions, and around the craniotomy site. Signals were recorded using a Cascade IOMAX system (Cadwell IONM Systems). The depth electrode was referenced to a vertex scalp electrode (Cz or Pz, depending on the approach), and activity was monitored in monopolar and bipolar montages together with simultaneous scalp EEG. Signals were sampled at 1000 Hz, band-pass filtered between 1 and 300 Hz, and visually inspected for IEDs by an experienced neurophysiologist. All recordings were obtained under general anesthesia, after cessation of muscle relaxants and reduction of anesthetic depth to avoid burst suppression.

Pre-resective iECoG sampling along the planned resection margins was used to characterize the baseline morphology and distribution of IEDs, which then served as a reference for subsequent intraoperative assessments. During resection, the depth electrode was kept in situ to guide the depth of tissue removal, while ioUS was repeatedly employed to verify anatomical resection boundaries and exclude residual pathology. Post-resective iECoG was systematically performed along the cavity walls, guided by real-time ioUS visualization. Persistence or modification of IEDs was interpreted intraoperatively by the multidisciplinary team in relation to pre-resection activity, ultrasound appearance, the pre-operative surgical plan, lesion type, and anticipated functional risk. When residual activity was considered concordant with the presumed EZ and surgically accessible without unacceptable morbidity, resection was cautiously extended and iECoG repeated. Conversely, when residual discharges were judged non-localizing, discordant, or when the expected surgical risk was deemed to outweigh the potential clinical benefit, no further resection was undertaken.

Postoperative CT was obtained within 6 h to exclude complications, and MRI at 6 months to confirm complete resection.

**Fig. 1:**
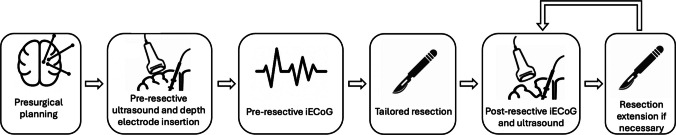
Surgical workflow integrating depth-electrode iECoG and ioUS guidance. After presurgical planning of resection margins and electrode trajectories using neuronavigation, pre-resective ioUS and depth-electrode insertion are performed to confirm target localization under real-time guidance. Pre-resective iECoG recordings direct tailored cortical and subcortical resection. Post-resective iECoG and ioUS are then used to verify the disappearance or modification of IEDs and to confirm resection completeness. When residual activity or lesion remnants are detected, the resection may be extended following multidisciplinary team discussion, with subsequent reassessment performed until final verification is obtained

The workflow from preoperative planning to resection strategy is illustrated in Fig. 1.

### Statistical analysis and Ethics

Given the small cohort size, results were summarized using descriptive statistics. Continuous variables are reported as median and interquartile range (IQR). Seizure outcomes were classified according to Engel and ILAE scales.

The study was approved by the institutional Ethics Committee and conducted in accordance with the Declaration of Helsinki. Given the retrospective design, the requirement for written informed consent was waived by the Ethics Committee. This study adheres to the PROCESS guidelines for reporting surgical case series [1].

## Results

Six patients with focal DRE underwent iECoG- and ioUS-guided resective surgery between 2022 and 2024. Demographic and clinical data are summarized in Table 1.

**Table 1:** Demographic and clinical characteristics of the study population. For each patient, key anatomo-electro-clinical findings are reported, along with the specific rationale for performing iECoG-guided resection

Pts	Age at seizure onset	Duration of epilepsy (years)	Age at surgery (years)	Seizure type and semeiology	scalp-EEG findings	Seizure frequency	MRI findings	FDG-PET findings	Rationale for iECoG-guided resection	Surgical procedure	Histopathology	Follow-up duration	Seizure outcome
1	13	26	39	**Focal impaired awareness seizure with observable manifestations** **Semiology:** Fear or terror aura followed by impaired awareness, laughter, and autonomic signs (tachycardia, apnea)	**Interictal**: negative **Ictal**: Diffuse bilateral rhythmic slow-wave discharges with superimposed fast activity, predominant in fronto-central regions and spreading to left temporal derivations	Multi-daily	Left ACC lesion	Hypomethabolism corresponding to MRI lesion	Deep-seated, non-well-defined EZ	Extendend lesionectomy	FCD IIb	24 months	ILAE1, ENGEL Ia
2	23	43	66	**Focal impaired awareness seizure with observable manifestations** **Semiology:** Bilateral dystonic posturing followed by loss of awareness and hypermotor behavior	**Interictal** right temporo-parietal spikes **Ictal**: Diffuse flattening predominant over the right parietal region, partially masked by movement artifacts	Daily	Right angular gyrus lesion, suggestive of FCD type II	Hypomethabolism corresponding to MRI lesion	- Long seizure history and diffuse ictal discharges: would suggest SEEG study - Age: suggested surgery as first option	Extended lesionectomy	FCD IIb	27 months	ILAE 5, ENGEL IVB
3	31	12	43	**Focal impaired awareness seizure with observable manifestations** **Semiology:** Dizziness, rising heat sensation, and transient speech impairment, followed by impaired awareness and postictal aphasia	**Interictal**: left fronto-temporal epileptiform acitivity **Ictal**: Diffuse desynchronization, prominent in left temporal leads, followed by sharp theta bursts in left temporal, suprasylvian, and vertex regions with subsequent contralateral propagation	Daily	two left temporal pole encephaloceles	Left temporal pole and uncus hypomethabolism	Evaluation of encephaloceles and hippocampal electrophysiological activity	Lesionectomy alone	Gliosis	20 months	ILAE1, ENGEL Ia
4	8	28	36	**Focal preserved awareness seizure with observable manifestations** **Semiology:** Rising heat from chest to throat, left upper-limb tingling, followed by hyperkinetic movements, automatisms, left-sided stiffness, and speech arrest	**Interictal**: Frequent theta activity intermixed with fast rhythms over bilateral fronto-centro-parietal channels, and small spikes over fronto-polar leads increasing during sleep **Ictal**: Low-voltage theta activity with small spikes over right fronto-polar, central, and vertex regions, occasionally spreading to contralateral homologous areas	Weekly	Right F1-F2 lesion, suggestive of FCD type II	Hypomethabolism corresponding to MRI lesion	Non-well-defined EZ	Lesionectomy alone	FCD IIb	40 months	ILAE 1; *ENGEL Id*
5	8	14	22	**Focal impaired awareness seizure with observable manifestations** **Semiology:** Visual misperception, followed by impaired awareness, mydriasis, and jargon aphasia	**Interictal**: Right anterior temporal spikes **Ictal**: Rhythmic theta activity arising from right fronto-temporal regions	Monthly/weekly	Diagnosis of tuberous sclerosis. Hyperintense stripes in FLAIR/T2 sequences in right temporopolar region and frontobasal region, extended to ventricular walls. Small subependymal nodules along lateral ventricles	Hypomethabolism in right lateral and mesial temporal regions	Decision whether to resect both tubers and/or extend the resection to temporomesial structures	Lesionectomy alone	Gliosis	12 months	ILAE 1, ENGEL Ia
6	39	6	45	**Focal impaired awareness seizure with observable manifestations** **Semiology:** Fluctuating impairment of verbal comprehension, speech production, and awareness, lasting up to 30 min and often evolving to bilateral tonic–clonic seizures	**Interictal**: Sharp waves and spike–wave discharges over left frontal leads **Ictal**: epileptiform activity originating in the left frontal channels and spreading to controlateral homologous regions	2–3/year	Left frontal gliotic scar as postoperative sequelae of arachnoid cyst marsupialization	Left frontomesial hypomethabolism corresponding to gliotic tissue	Deep-seated, non-well-defined EZ	Lesionecomy alone	Arachnoid cyst (gliosis, chronic histiocytic inflammatory infiltrate, and hemosiderin deposits)	12 months	ILAE 1, ENGEL Ia

Age at seizure onset ranged from 8 to 39 years (median 18; IQR 8–31), with epilepsy duration ranging from 6 to 43 years (median 20; IQR 12–28). Age at surgery ranged from 22 to 66 years (median 41; IQR 36–45). Seizure frequency ranged from multiple daily episodes to 2–3 per year. MRI revealed structural abnormalities in all patients, including FCDs, encephaloceles, a gliotic postoperative scar, and tuberous sclerosis–related lesions. Neuroimaging post-processing was successfully applied in all cases to support surgical planning. PET–MRI fusion confirmed hypometabolic changes concordant with the MRI-defined lesion in every patient. In those with suspected focal cortical dysplasia, MAP analysis further supported lesion localization. No relevant discrepancies between conventional MRI and post-processing outputs were observed.

Based on presurgical anatomo-electro-clinical findings, the EZ was localized to the frontal lobe in four patients, the temporal lobe in one, and the parietal lobe in another.

Combined ioUS and iECoG guidance was feasible in all procedures. Pre-resective ioUS provided reliable lesion visualization and confirmed electrode placement in every case. All lesion appeared clearly hyperechoic, corresponding to the MRI T2-hyperintense abnormality. Pre- and post-resective iECoG sampling detected IEDs in four (66%) patients, leading to resection extension in two. No intraoperative complications occurred, and all resections were completed as planned.

At last follow-up (6–40 months, median 22; IQR 12–27), five of six patients were seizure-free (ILAE 1, Engel I).

Postoperative CT showed no electrode- or resection-related complications. Across the series, a total of 37 depth-electrode insertions were performed (minimum 3, maximum 8, mean 6 per patient), counting both pre- and post-resection sampling trajectories. MRI at 6 months confirmed complete lesion removal in all patients, and no new neurological deficits were observed at discharge or 6-month follow-up.

Additional operative time related to the workflow was retrospectively estimated. Depth-electrode iECoG assessment required approximately 20 min before and 20 min after resection, while iterative ioUS scans added 10 min in total, largely without disrupting the surgical workflow.

In both cases where resection was extended based on iECoG findings (Tab. 1, Patients 1 and 4), histopathology revealed FCDs. One lesion was located in the anterior cingulate cortex and the other in the lateral frontal cortex.

In the patient with tuberous sclerosis complex (Tab. 1, Patient 5), removal of the frontal tuber interestingly modulated the electrophysiological activity recorded in the temporal tuber and subsequent resection of the latter resulted in disappearance of residual IEDs.

In another patient (Tab. 1, Patient 2), ioUS confirmed gross-total resection of a right angular FCD, but post-resective iECoG revealed residual IEDs inconsistent with the pre-resection pattern. Given the uncertain significance of these findings and the patient’s long-standing epilepsy, resection was not extended. This was the only non–seizure-free case (Engel IVB, ILAE 5).

In the remaining two patients, no clear intraoperative epileptiform activity was recorded. However, the absence of pre-resective IEDs after repeated samplings prompted intraoperative re-discussion of the surgical plan. In one case (Tab. 1, Patient 3), involving dual left temporal-pole encephaloceles and long-standing mesial temporal lobe epilepsy, the lack of hippocampal spikes supported a hippocampal-sparing approach, with the patient remaining Engel IA after 24 months. The other case (Tab. 1, Patient 6) involved a left frontal gliotic scar after arachnoid cyst marsupialization; absence of epileptiform discharges in the gyri underlying the scar margins prompted a limited resection. The patient has remained seizure-free at the 12-month follow-up.

### Illustrative case (Table 1, Patient 1)

A 39-year-old woman presented with drug-resistant epilepsy, misdiagnosed as panic disorder since age 13. At age 38, seizures were correctly attributed to anterior cingulate cortex (ACC) epilepsy. Clinically, episodes consisted of brief (approximately 10 s) aura with fear or euphoria, tachycardia, apnea, occasionally progressing to impaired awareness with laughing, verbigeration, and dyspnea. Seizures occurred multiple times daily and were unresponsive to clobazam and lacosamide.

**Fig. 2:**
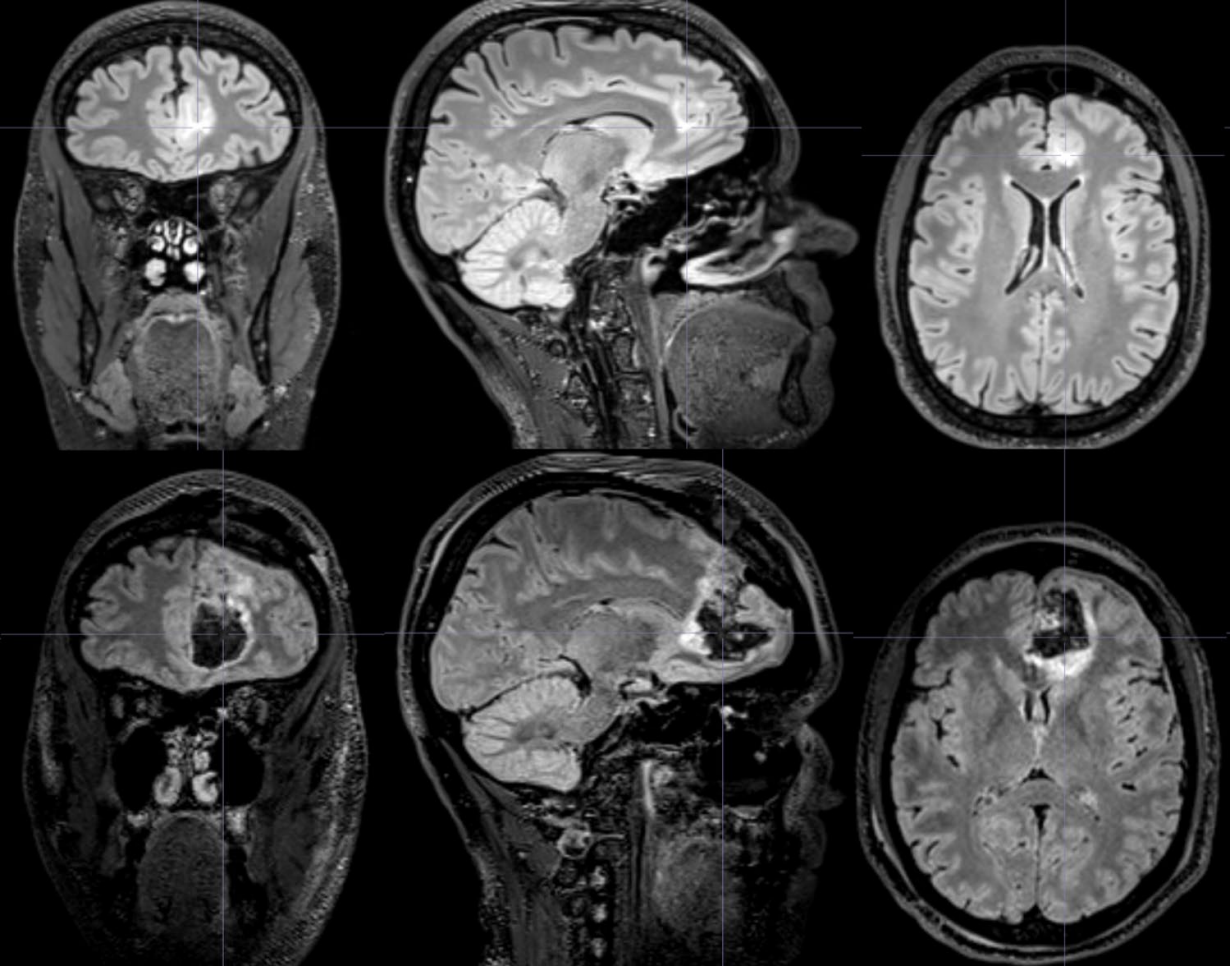
Pre- and postoperative imaging. (Upper row) Coronal, sagittal, and axial T2-FLAIR MRI slices showing a hyperintense lesion in the left anterior cingulate cortex. (Lower row) Postoperative T2-FLAIR images confirming the extended lesionectomy

Video-EEG monitoring documented left fronto-central seizure onset with rapid bilateral propagation. MRI revealed a stable cortical-subcortical lesion in the left ACC, hyperintense on T2, without contrast enhancement (Fig. 2), corresponding to a region of hypometabolism on FDG-PET. A presumptive diagnosis of glioneuronal tumor or FCD was made.

**Fig. 3:**
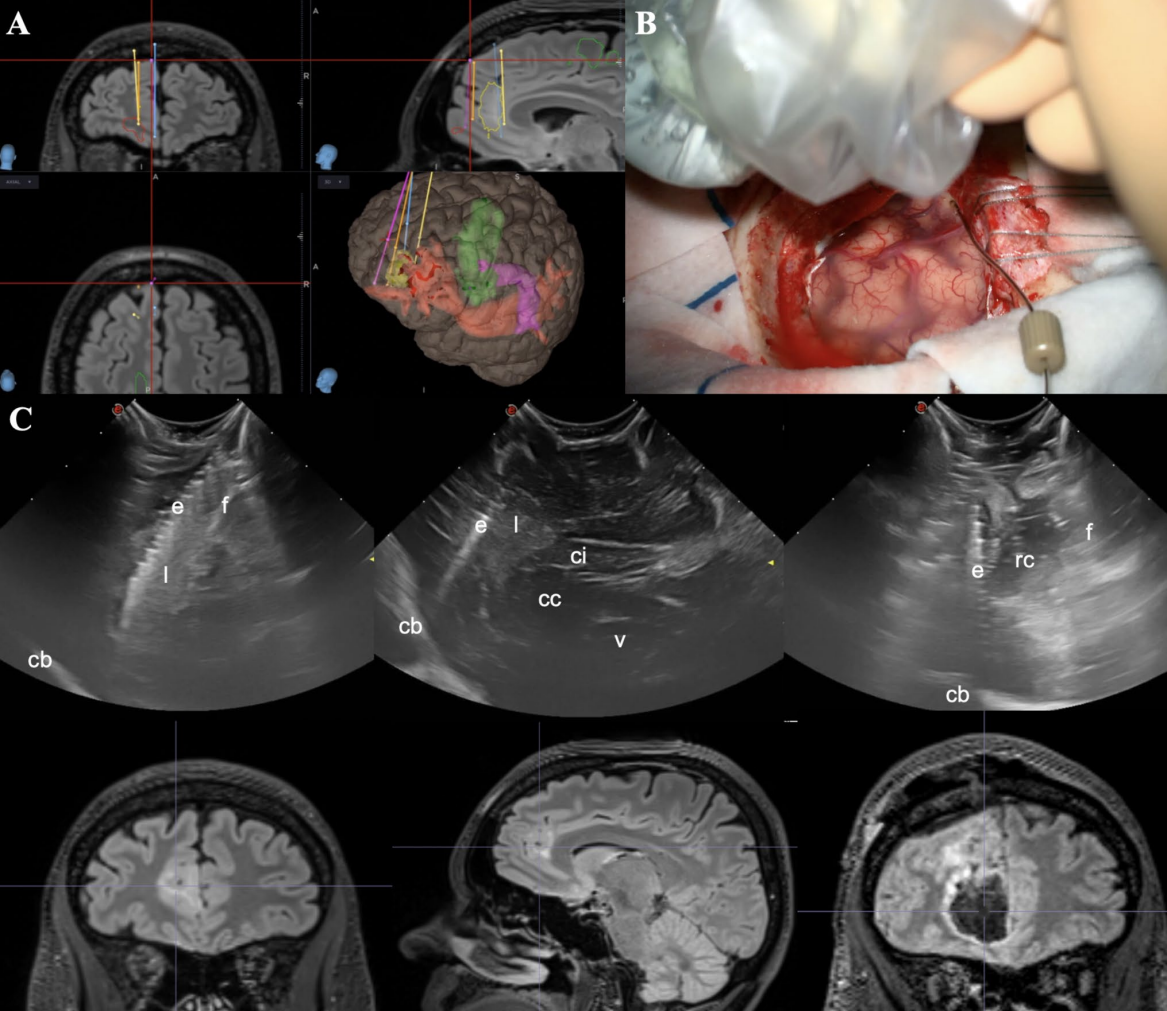
Presurgical neuronavigation planning and depth electrode positioning with ioUS. (Panel A) Preoperative neuronavigation planning, including 3D reconstructions of the lesion (yellow), major white matter tracts (frontal aslant tract, green; inferior fronto-occipital fasciculus, red; arcuate fasciculus, violet), and planned trajectories for intracerebral depth-electrode iECoG exploration. (Panel B) Intraoperative ioUS acquisition over the depth electrode positioned at the anteromesial border of the lesion. (Panel C) ioUS images compared with corresponding MRI cuts, flipped into anatomical orientation for better comprehension. Coronal and sagittal ioUS views show the electrode within the hyperechogenic lesion and its relationship with the falx cerebri and corpus callosum. The last coronal ioUS image, obtained after resection, depicts the electrode lateral to the resection cavity during post-resective iECoG sampling. Abbreviations: cb, cranial base; cc, corpus callosum; ci, cingulate gyrus; e, electrode; f, falx cerebri; l, lesion; rc, resection cavity; v, ventricle

**Fig. 4:**
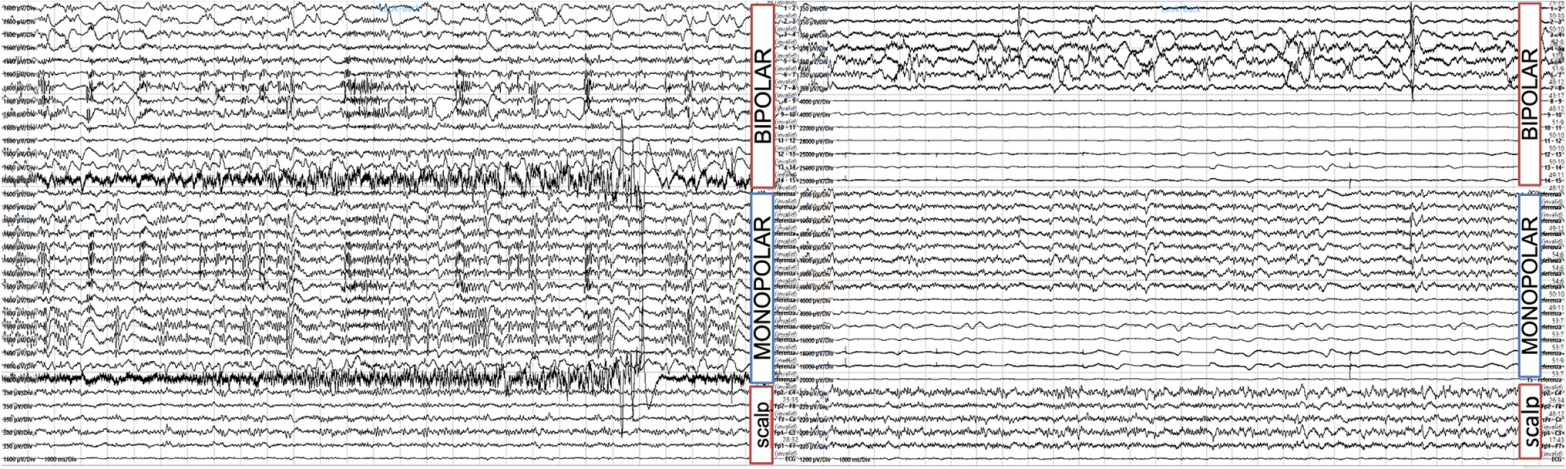
Intraoperative depth-electrode iECoG recordings before and after lesionectomy. On the left, pre-lesionectomy iECoG recording: a 15-contact depth electrode was placed within the lesion. In both bipolar and monopolar montages, contacts 5–9 showed absence of background activity with frequent polyspike complexes and no clear correlation on scalp EEG. On the right, post-lesionectomy iECoG recording: a 15-contact depth electrode was placed in the mesial-posterior portion of the resection cavity (first seven contacts). Diffuse slowing was observed in both bipolar and monopolar montages (contacts 3–7), with occasional spikes on contacts 3–5 and no clear correlation on scalp EEG

The patient underwent lesionectomy guided by iECoG, ioUS and neuronavigation (Fig. 3). Depth-electrode recordings precisely mapped epileptiform discharges along the lesion margins, guiding resection extent. ioUS facilitated accurate electrode positioning and confirmed lesion boundaries in real time. Post-resection iECoG revealed residual spiking, prompting further targeted resection until complete electrophysiological normalization was achieved (Fig. 4).

The postoperative course was uneventful, with no neurological deficits. Histopathology confirmed a FCD type IIb.

## Discussion

This study describes the application of iECoG combined with ioUS to guide tailored resection in patients with DRE and MRI-visible lesions. In all included cases, preoperative evaluation identified a structural lesion and yielded concordant anatomo–electro–clinical data indicating a single corresponding epileptogenic focus, such that SEEG was not expected to provide additional diagnostic value given its intrinsic limitations in spatial sampling. Yet, structural abnormalities do not always correspond to the true spatial extent of the EZ. This mismatch may result from indistinct lesion margins or the presence of epileptogenic tissue that appears structurally normal on imaging and intraoperatively. Within this context, the present report provides, to our knowledge, the first structured description of a real-world workflow for depth-electrode iECoG-ioUS-guided resective epilepsy surgery. This approach allows extending the resection beyond the visible abnormality when indicated, or verification of its completeness despite a macroscopically normal-appearing surrounding cortex. The integration of ioUS and depth-electrode iECoG may therefore represent a pragmatic way to bridge the gap between intraoperative anatomical and functional delineation of the EZ in selected MRI-positive cases.

### iECoG findings and implications

In all cases, iECoG sampling contributed to intraoperative definition of the resection plan. In four patients (Tab. 1; Patients 1, 2, 4, and 5), intraoperative detection of interictal discharges directly influenced surgical strategy. Two resections were extended due to persistent activity, while in another the uncertain significance of residual abnormal discharges discouraged further resection. This finding anticipated the unfavorable outcome, as this patient did not achieve seizure freedom. In two patients, no clear IEDs were detected intraoperatively. In one, negative sampling allowed preservation of the hippocampal formation by limiting resection to two temporal encephaloceles; in the other, involving an epileptogenic frontal gliotic scar, the absence of extra-lesional activity supported the decision to proceed with lesionectomy alone. At last follow-up, five of six patients achieved seizure freedom (ILAE 1, Engel I).

The present series illustrates the heterogeneous clinical contexts in which intraoperative iECoG can be effectively applied. Cases included both classic MRI lesions, such as FCDs or tubers with uncertain functional boundaries or subcortical extension, and atypical entities, including encephaloceles and gliotic scars, in which the epileptogenic relevance of the lesion was uncertain.

These findings support the feasibility and potential utility of this multimodal intraoperative strategy in tailoring resective epilepsy surgery. The combined use of ioUS and depth iECoG provided complementary real-time anatomical and electrophysiological feedback, enhancing intraoperative decision-making.

### Evidence and technical insights

Two recent systematic reviews and meta-analyses evaluated the impact of iECoG-guided resections on seizure outcomes [15, 17]. One identified a potential benefit at the individual patient data (IPD) level but found no significant differences between patients with positive or negative post-resection iECoG when analyzed at both IPD and study levels [15]. Conversely, the other review supported resecting all cortex exhibiting IEDs to improve seizure control [17]. However, key questions remain regarding whether the benefit of iECoG is pathology-dependent and whether outcomes differ between temporal (particularly in hippocampal sclerosis) and extratemporal resections. Current evidence is limited to retrospective series, and the only available randomized trial compared IED- with HFO-guided resections. Nevertheless, that study did not assess outcomes of procedures performed with versus without iECoG guidance, precluding a definitive evaluation of its clinical impact [28].

Importantly, patients selected for iECoG-guided resections often present with complex scenarios, such as ill-defined EZs or proximity to eloquent cortex, which inherently increase the risk of incomplete resection and may bias outcome data. Another major limitation in the literature is the absence of standardized protocols for surgical planning and iECoG interpretation. Incorporating advanced preoperative post-processing tools, such as the Morphometric Analysis Program (MAP), may enhance EZ delineation even independently of iECoG, or in synergy with it [13, 29].

In our approach, the choice to use depth electrodes instead of subdural strips allowed sampling from cortical regions not accessible to surface electrodes, including deep sulcal fundi and buried gyri. In such cases, subdural electrodes may capture propagated rather than primary local activity, potentially leading to inaccurate localization of the EZ. To date, no direct comparison between depth- and strip-based iECoG has been reported, and future studies should determine whether additional sulcal sampling translates into more accurate EZ delineation and, ultimately, improved seizure outcomes.

The added value of ioUS was twofold: it enabled accurate targeting of suspected lesions and optimized both electrode placement and surgical orientation. Furthermore, ioUS mitigates the effects of brain shift and deformation, which may limit neuronavigation accuracy, providing reliable real-time anatomical guidance [14].

### Limitations

The main limitations of this study are the small cohort size and its heterogeneity. Nonetheless, our aim was to illustrate the technical feasibility, safety, and potential clinical utility of this intraoperative strategy across a spectrum of real-world scenarios. Additional constraints are intrinsic to iECoG itself: surgical strategies vary widely, ranging from resection of all cortex exhibiting interictal spikes to limiting resection to structural lesions, particularly when spiking involves eloquent regions. Moreover, recorded electrophysiological activity may be influenced by anesthesia and can originate from adjacent but non-epileptogenic tissue, complicating interpretation. No consensus currently exists on the degree of residual spiking that can be considered acceptable. Finally, iECoG increases operative time, requires specialized expertise, and poses organizational challenges. Its application should therefore be individualized, taking into account the lack of standardized interpretation criteria and the variability of protocols across centers. In this case series, additional operative time was not prospectively recorded, and the figures reported represent retrospective estimates. Future work should formally assess the time impact of this workflow.

iECoG differs fundamentally from SEEG, which allows prolonged sampling in the awake state and wide cortical exploration. Accordingly, our results should not be interpreted as suggesting that iECoG can replace SEEG. Instead, iECoG may complement intraoperative decision-making only in carefully selected MRI-positive cases where SEEG is not clinically indicated a priori.

The integration of depth-electrode iECoG with ioUS represents a refined surgical strategy, but robust evidence is needed to definitively establish its efficacy and to optimize its use. In addition, our favorable outcomes may also reflect the intrinsic prognostic advantage of MRI-positive epilepsy with concordant anatomo-electro-clinical localization, together with the contribution of systematic multimodal presurgical evaluation.

## Conclusion

This case series provides the first structured, real-world description of a workflow integrating depth-electrode iECoG with ioUS during resective epilepsy surgery. By combining complementary real-time electrophysiological and anatomical feedback, this approach may assist intraoperative delineation of the EZ and support surgical decision-making in selected MRI-positive cases where SEEG is not indicated.

While these preliminary results are encouraging, further prospective and multicenter studies, ideally including controlled or randomized comparative designs, are needed to determine whether the integration of iECoG and ioUS can translate into measurable improvements in surgical precision and long-term seizure outcomes.

## Data Availability

No datasets were generated or analysed during the current study.
